# Molecular Evolution of RNA-Dependent RNA Polymerase Region in Norovirus Genogroup I

**DOI:** 10.3390/v15010166

**Published:** 2023-01-05

**Authors:** Nan Zhou, Yue Huang, Lu Zhou, Mingma Li, Hui Jin

**Affiliations:** 1 Key Laboratory of Environmental Medicine and Engineering of Ministry of Education, Department of Epidemiology and Health Statistics, School of Public Health, Southeast University, Nanjing 210009, China; 2 Department of Acute Infectious Diseases, Jiangsu Provincial Center for Disease Control and Prevention, Nanjing 210009, China

**Keywords:** norovirus GI, RNA-dependent RNA polymerase, evolution

## Abstract

Norovirus is the leading viral agent of gastroenteritis in humans. RNA-dependent RNA polymerase (RdRp) is essential in the replication of norovirus RNA. Here, we present a comprehensive evolutionary analysis of the norovirus GI RdRp gene. Our results show that the norovirus GI RdRp gene can be divided into three groups, and that the most recent common ancestor was 1484. The overall evolutionary rate of GI RdRp is 1.821 × 10^−3^ substitutions/site/year. Most of the amino acids of the GI RdRp gene were under negative selection, and only a few positively selected sites were recognized. Amino acid substitutions in the GI RdRp gene accumulated slowly over time. GI.P1, GI.P3 and GI.P6 owned the higher evolutionary rates. GI.P11 and GI.P13 had the faster accumulation rate of amino acid substitutions. GI.P2, GI.P3, GI.P4, GI.P6 and GI.P13 presented a strong linear evolution. These results reveal that the norovirus GI RdRp gene evolves conservatively, and that the molecular evolutionary characteristics of each P-genotype are diverse. Sequencing in RdRp and VP1 of norovirus should be advocated in the surveillance system to explore the effect of RdRp on norovirus activity.

## 1. Introduction

Norovirus belongs to the family *Caliciviridae* and the genus *Norovirus*. Its genome is a positive-sense, single-stranded RNA with the length of 7.5 kb, encoding three open reading frames (ORFs) [1]. ORF1 encodes a large polyprotein consisting of six nonstructural proteins (designated as NS1/2 to NS7), and one of these is RNA-dependent RNA polymerase (RdRp) [2]. ORF2 encodes the major structural protein, VP1, including shell (S) and protruding (P) domains, and is the major epitope for neutralization antibody [3,4]. ORF3 encodes a minor structural protein, VP2 [5]. According to the diversity of the VP1 protein, norovirus can be classified into 10 genogroups (GI~GIX), and GI, GII, GIV, GVIII and GIX can infect humans [6]. Norovirus GI and GII are the leading viral agents for human gastroenteritis, and about 16% of gastroenteritis cases were caused by them [7]. More importantly, norovirus GII is the leading viral agent in gastroenteritis outbreak [8,9,10,11]. The economic burden associated with norovirus in societal costs per year was up to USD 60 billion [12].

Norovirus has a high genetic-diversity, and more than 9 and 26 genotypes in GI and GII genogroup are identified based on the VP1 gene, respectively [6]. Serval variants can be further recognized within some genotypes. For example, more than six GII.4 variants were observed, and an emerged variant would induce an increase in norovirus activity [13]. In the winter of 2014, an emerging GII.17 variant became more prevalent in the population [14].

The evolution of norovirus is driven by the accumulation of point mutation and recombination. The hotspot region of norovirus recombination is located at the junction of ORF1 and ORF2, making the genome acquire a new RdRp fragment [15]. A dual-nomenclature system was accordingly established by the International Norovirus Working Group based on the VP1 and RdRp gene [16]. Recently, 14 and 37 P-genotypes in the GI and GII RdRp region were identified, respectively [6], and NoroSurv data showed that most of the dual-typed sequences from 2016 to 2020 were recombination strains, including GII.4P16, GII.4P31, GII.6P7, GI.3P13, GI.5P4 and so on [17]. Furthermore, some studies have presented that the epidemic of some genotypes or variants, such as GII.4 and GII.2, was associated with the acquisition of a new RdRp [18,19]. The phylogenetic feature of GII.3 VP1 strains was related to a different ORF1 genotype [13].

The synthesis of norovirus RNA is modulated by an interaction between RdRp and VP1 [20], and RdRp plays a significant role in the norovirus life cycle. Thus, molecular evolutionary analysis of the RdRp gene is essential to understand the circulation of norovirus in humans. Recently, RdRp of the norovirus GII was analyzed by Ozaki et al. [2]. Recombination in norovirus GI is also common [21]; however, there is no detailed evolutionary analysis in the norovirus GI RdRp region. Here, we provided a comprehensive analysis of evolutionary characteristics in the norovirus GI RdRp gene by analyzing the full-length gene.

## 2. Materials and Methods

### 2.1. Dataset

Full-length nucleotide strains of norovirus GI RdRp were retrieved from the GenBank database (accessed on 15 September 2022) and were genotyped by a norovirus genotyping tool [22]. Strains with an unknown collection year, undetermined nucleotides, or from environmental samples were excluded. Additionally, strains with recombination signals detected via Recombination Detection Program (RDP) version 4.56 by more than three methods with the *p*-value threshold of 0.05 were also excluded [23].

### 2.2. Genetic and Amino Acid Diversity

Sequence alignment and identity analysis were conducted by BioEdit 7.1.3.0 [24]. The intergenotype mean amino acid distances were calculated by MEGA 7.0.26 using a Poisson model [25]. Furthermore, amino acid position with greater variability was recognized by Shannon’s entropy value estimated by the web service of the Shannon Entropy-One tool (www.hiv.lanl.gov (accessed on 20 September 2022)). The phylogenetic tree of norovirus GI RdRp was constructed by the maximum likelihood (ML) method using MEGA 7.0.26 with 1000 bootstrap replications, and the best-fit nucleotide substitution model was estimated according to a Bayesian Information Criterion (BIC) score by the IQ-TREE web server [26]. The pairwise phylogenetic distances were estimated based on an ML tree using Patristic software [27].

### 2.3. Root-to-Tip Divergence Analysis

TempEst v1.5 was used to infer the root-to-tip divergence based on an ML phylogenetic tree [28]. Then, the root-to-tip divergence was plotted against the collection time of strain, and a linear regression analysis was also conducted to evaluate the evolutionary clock-like pattern of the norovirus GI RdRp gene.

### 2.4. Accumulation Pattern of Amino Acid Substitutions

A previously described algorithm was used to demonstrate the accumulation pattern of amino acid substitutions of the norovirus GI RdRp gene [29,30]. In brief, the number of pairwise amino acid differences was calculated by MEGA 7.0.26 and averaged. Then, the mean number of pairwise amino acid differences was plotted against the timespan of isolation to discuss the accumulation pattern of amino acid. Parameters of linear regression were estimated to evaluate the possible linear accumulation and the accumulative rate.

### 2.5. Selection Pressure Analysis

Selection pressure analysis was conducted on the Datamonkey server via fixed effects likelihood (FEL), internal fixed effects likelihood (IFEL), single-likelihood ancestor counting (SLAC) and MEME methods [31]. A positively selected site was defined as the nonsynonymous (dN) > synonymous (dS) substitutions ratio, and a negatively selected site was defined as the dN < dS. The *p*-value threshold was 0.05. Then, positively selected sites and sites with greater variability were mapped onto a 3D structure of RdRp protein (PDB number: 1SH0) by PyMOL version 2.3.0 [32].

### 2.6. Estimation of Evolutionary Parameters

Evolutionary rate and the most recent common ancestor (TMRCA) were estimated based on the Bayesian Markov Chain Monte Carlo (MCMC) method in the BEAST package 2.0 [33]. The best-fit nucleotide substitution model was determined by the IQ-TREE web server as described. The MCMC chain was run on the length of 100,000,000 steps. Three clock models (strict clock, relaxed clock exponential and relaxed clock log normal) and two tree prior models (coalescent constant population and coalescent exponential population) were selected and compared by Akaike’s Information Criterion through MCMC (AICM) using Tracer version 1.6 (http://tree.bio.ed.ac.uk/software/tracer/ (accessed on 28 September 2022)) [34].

## 3. Results

### 3.1. Description of Dataset

A total of 205 norovirus GI RdRp strains were included in our analysis, ranging from 1968 to 2021 (Supplementary Table S1). These strains belonged to 14 P-genotypes (GI.P1~GI.P14), and GI.P3 (18.54%, 38/205), GI.P1 (16.10%, 33/205), GI.P11 (14.63%, 30/205) and GI.P13 (9.76%, 20/205) were the four most predominant genotypes. Each included strain had the VP1 genotype, and a recombination event was observed in GI.3P10, GI.3P13, GI.3P14, GI.5P12, GI.5P4 and GI.6P11.

### 3.2. Diversity of Norovirus GI RdRp Gene

Nucleotide and amino acid identities of the GI RdRp gene were 70.5~100% and 81.2~100%, respectively. For each P-genotype, the higher heterogeneity at the nucleotide identity level was observed in GI.P1, GI.P3, GI.P7 and GI.P13 (Table 1). At the amino acid level, GI.P1 and GI.P3 had higher heterogeneity. The intergenotype mean amino acid distances ranged from 0.019 to 0.198. The minimum value was between GI.P5 and GI.P12, and the maximum value was between GI.P1 and GI.P14 (Supplementary Table S2). In order to recognize the high-variable amino acid site of an intragenotype, entropy values of amino acid site for each P-genotype with more than 10 strains were calculated, and the high-variable site was determined as an entropy value greater than 0.6. Our results showed that there was no high-variable amino acid site in GI.P2, GI.P4 or GI.P6. More than two high-variable sites were identified in GI.P1 (at position 17, 137, 141, 174, 192, 437 and 497), GI.P3 (at position 120, 380 and 437), GI.P7 (at position 76, 120, 133, 267, 273, 355, 382, 480 and 491), GI.P11 (at position 383 and 437) and GI.P13 (at position 1, 17, 48, 106, 155, 423 and 433). Furthermore, position 17 was the high-variable site in GI.P1 and GI.P13, and position 120 was the high-variable site in GI.P3 and GI.P7. Position 437 was recognized as a high-variable site in three P-genotypes (GI.P1, GI.P3 and GI.P11).

### 3.3. Phylogenetic Analysis of Norovirus GI RdRp Gene

**Figure 1:**
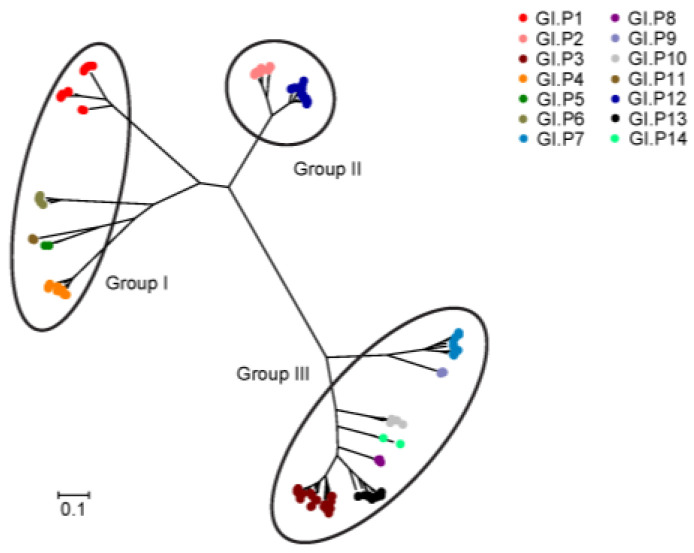
Molecular phylogenetic analysis of norovirus GI RdRp gene. This phylogenetic tree was constructed by the maximum likelihood method based on the general time reversible model.

Phylogenetic analysis was constructed based on the ML method. The ML phylogenetic tree showed that norovirus GI RdRp strains can be divided into three groups (Figure 1). Group I consists of GI.P1, GI.P4, GI.P5, GI.P6 and GI.P12. Group II contains GI.P2 and GI.P11. Group III includes GI.P3, GI.P7, GI.P8, GI.P9, GI.P10, GI.P13 and GI.P14. The pairwise phylogenetic distances based on the ML method among all GI RdRp strains were from 0 to 0.18257 and averaged 1.126 ± 0.570 (mean ± SD). We also conducted the phylogenetic analysis in the P-genotypes with more than 10 strains. Our results revealed that except for GI.P2 and GI.P13 presenting two variants, GI.P1, GI.P3, GI.P4, GI.P6, GI.P7 and GI.P11 can be grouped into three variants (Supplementary Figures S1–S8). GI.P1 (0.169 ± 0.166), GI.P3 (0.118 ± 0.060) and GI.P7 (0.119 ± 0.060) had the highest mean pairwise phylogenetic distance at the intragenotype level (Supplementary Table S3).

### 3.4. Root-to-Tip Divergence Analysis

**Figure 2:**
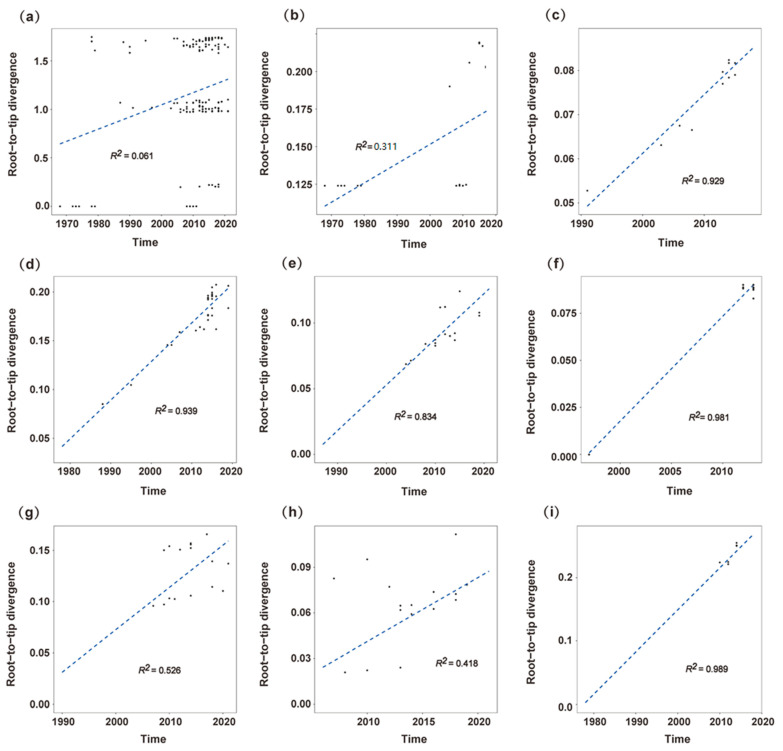
Root-to-tip divergence plots of norovirus GI RdRp gene: (**a**) all strains, (**b**) GI.P1, (**c**) GI.P2, (**d**) GI.P3, (**e**) GI.P4, (**f**) GI.P6, (**g**) GI.P7, (**h**) GI.P11, (**i**) GI.P13. The X-axis represents the isolation time, and the Y-axis is the root-to-tip divergence inferred from maximum likelihood tree. Blue dashed line means a linear regression line.

Root-to-tip divergence plots based on ML trees in all included GI RdRp strains and genotypes with more than 10 strains were conducted. Our results showed that the GI RdRp gene evolved with a poor clock-like signal, and that the coefficient of determination (R^2^) value was 0.063 (Figure 2a). For each genotype, GI.P1, GI.P7 and GI.P11 presented a moderate clock-like evolution with R^2^ values of 0.311, 0.526 and 0.418, respectively (Figure 2b,g,h). GI.P2, GI.P3, GI.P4, GI.P6 and GI.P13 presented a strong linear evolution with R^2^ values of 0.929, 0.939, 0.834, 0.982 and 0.989 (Figure 2c–f,i).

### 3.5. Accumulation Pattern of Amino Acid Substitutions

**Figure 3:**
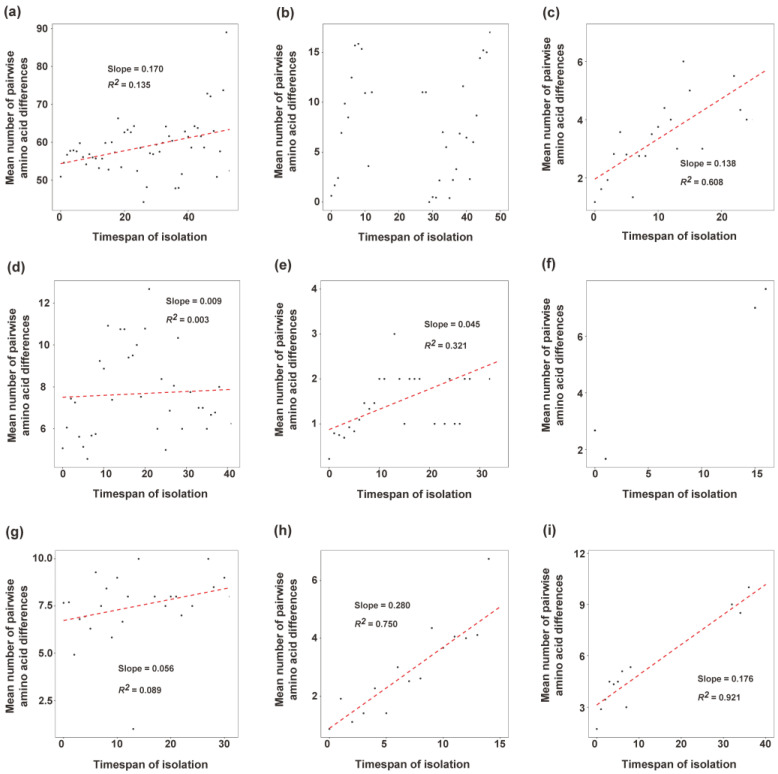
Amino acid accumulation pattern of norovirus GI RdRp gene: (**a**) all strains, (**b**) GI.P1, (**c**) GI.P2, (**d**) GI.P3, (**e**) GI.P4, (**f**) GI.P6, (**g**) GI.P7, (**h**) GI.P11, (**i**) GI.P13. The X-axis is the timespan of isolation, and the Y-axis indicates the mean number of pairwise amino acid differences. Red dashed line shows a linear regression line.

We plotted the mean pairwise amino acid differences against the timespan of isolation to evaluate the accumulation pattern of amino acid substitutions. Our results showed that amino acid substitutions in the GI RdRp gene accumulated slowly over time (Figure 3a) and presented a weak linear trend (R^2^ = 0.135). For each genotype with more than 10 strains, GI.P3 and GI.P7 also presented weak linear trends (Figure 3d,g), and GI.P2 and GI.P4 presented moderate linear trends (Figure 3c,e). GI.P11 and GI.P13 presented strong linear accumulation (Figure 3h,i). GI.P11 and GI.P13 also owned the faster accumulation rate of amino acid substitutions (Slopes were 0.280 and 0.176, respectively), and the amino acid substitutions in GI.P3 and GI.P4 accumulated more slowly than other genotypes (slopes were 0.009 and 0.045, respectively). In GI.P1, timespan of isolation had no effect on the accumulation of amino acid substitutions (Figure 3b). Because there were only four points for analysis, the accumulation pattern of amino acid substitutions of GI.P6 was not evaluated (Figure 3f). 

### 3.6. Selection Pressure Analysis

**Figure 4:**
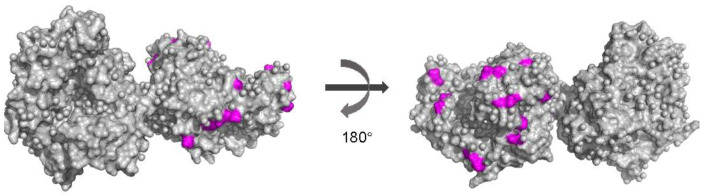
Mapping of positively selected and high-variable sites (colored in light magenta) on the structural model of norovirus GI RdRp protein (PDB number: 1SH0).

Selection pressure analysis was performed by the SLAC, MEME, FEL and IFEL methods. At the level of all included norovirus GI RdRp strains, more than 400 negatively selected sites were recognized by the SLAC, FEL and IFEL methods, but no positive selection site was observed by these methods, and only four sites were under positive selection (at positions 229, 315, 423 and 476) detected by the MEME method. For P-genotypes with greater than three strains, positively selected sites were also only observed in GI.P1 (at position 315), GI.P2 (at position 254) and GI.P13 (at positions 48 and 315) by the MEME method. Then, we mapped positively selected and high-variable sites on the 3D structure of RdRp protein, and these sites were surface-exposed and located at the same chain of the dimer structure (Figure 4).

### 3.7. Evolutionary Parameters of Norovirus GI RdRp Gene

For all included norovirus GI RdRp strains and P-genotypes with more than 10 strains, evolutionary rate and TMRCA were estimated. Our results showed that the overall evolutionary rate of the GI RdRp region was 1.821 × 10^−3^ (95% highest posterior densities (HPDs): 1.294 × 10^−3^–2.344 × 10^−3^) substitutions/site/year. GI.P3 owned the highest rate (4.303 × 10^−3^, 95% HPDs: 2.728 × 10^−3^–5.954 × 10^−3^ substitutions/site/year), and GI.P2 had the lowest rate (1.373 × 10^−3^, 95% HPDs: 8.409 × 10^−4^–1.896 × 10^−3^ substitutions/site/year). The evolutionary rates of GI.P4 and GI.P13 were similar (2.532 × 10^−3^ vs. 2.635 × 10^−3^). The year of divergence of the GI RdRp region was about 540 years ago, and GI.P1, GI.P2 and GI.P13 had a similar divergence year to each other (Table 2).

## 4. Discussion

Recently, several studies have reported the evolutionary characteristics of norovirus. For example, Kobayashi et al. studied the molecular evolution of the capsid gene in all genotypes of norovirus GI and GII [35,36]. Some specific genotypes, such as GII.2, GII.3, GII.4, GII.17, GI.3 and so on, were also evaluated [37,38,39,40,41]. However, these studies were limited to the norovirus VP1 gene. RdRp is one significant nonstructural protein in viral replication [42], and evolution of RNA viruses can accelerate due to the error-prone nature of RdRp [43]. Although there were some studies about norovirus RdRp, they focused on norovirus GII [44,45,46]. Evolutionary analysis in the norovirus GI RdRp region was absent. In this study, we downloaded full-length norovirus GI RdRp strains from the GenBank database to provide a comprehensive description of molecular characteristics on the norovirus GI RdRp region.

In our analyzed dataset, GI.P3 was the most dominant P-genotype, and this was consistent with the result from a comprehensive review, in which GI.P3 was also the most prevalent P-genotype of norovirus GI [47]. Phylogenetic analysis exhibited that the norovirus GI RdRp gene can be clustered into three groups. The norovirus VP1 gene was reported to contain two groups [35]. We compared the distribution of genotypes in phylogenetic groups between norovirus GI RdRp and VP1. The P-genotypes in phylogenetic group I and III of RdRp were relatively consistent with the G-genotypes in phylogenetic group I and II of VP1. This may suggest that the phylogenetic relationships of norovirus GI RdRp and VP1 are similar.

Then, we evaluated the genetic diversity of the norovirus GI RdRp gene. There is no report about the genetic diversity of the norovirus RdRp region besides one showing that the mean pairwise phylogenetic distance of the GII RdRp gene estimated by the ML method was 0.549 ± 0.486 [2], which was lower than our estimates in the GI RdRp region (1.13 ± 0.57). This reveals a higher genetic divergence of GI RdRp. At the intragenotype level, more than two variants were observed in the phylogenetic analysis, and identity and phylogenetic distance analysis revealed that GI.P1, GI.P3, GI.P7 and GI.P13 had higher variation. The diversity of norovirus VP1 capsid was partially associated with epidemiological patterns of genotype, and limited diversity of VP1 capsid indicated limited spatiotemporal predominance [48]. A meta-analysis showed that GI.3, GI.6, GI.4 and GI.5 were the significant norovirus GI genotypes in children with gastroenteritis [49], and these are not fully consistent with the diversity of the norovirus GI RdRp gene. Thus, the association between the diversity of the norovirus GI RdRp gene and the prevalence of norovirus GI in the population needs to be explored.

By analysis of the VP1 gene, Tohma et al. reported that non-GII.4 genotypes evolved linearly at the intravariant level [48]. We estimated the clock-like manner of evolution of the norovirus GI RdRp gene by root-to-tip divergence analysis. Our results showed that although root-to-tip temporal signal was not apparent in other P-genotypes, GI.P2, GI.P3, GI.P4, GI.P6 and GI.P13 presented strong linear evolution, which may mean that they evolved as a whole at the genotype level. However, we cannot draw a conclusion that the evolutionary clock-like patterns of norovirus GI RdRp and VP1 were different, since a root-to-tip divergence plot was only conducted in GI.3 in Tohma’s study. Additionally, a larger sample size is needed to analyze norovirus GI RdRp at the intravariant level.

According to the accumulation pattern of amino acids of capsid, Parra et al. already recognized two patterns of diversification of norovirus [50]. One is called “evolving”, in which the number of amino acid differences accumulated over time (GII.4 genotype), and one is called “static”, in which norovirus capsid presents conserved and amino acid differed by only a few residues over decades (non-GII.4 genotypes). There are no data on the accumulation pattern of amino acid of norovirus RdRp. In our work, we found that the accumulation of amino acid substitutions in norovirus GI RdRp was slower, and the mean amino acid difference in the timespan of 20 years only accumulated about 1%. Purifying selection can shape the viral evolution. In this study, most P-genotypes of norovirus GI showed no positively selected sites. This is similar with the norovirus GII RdRp gene [2]. RdRp involves the replication process of an RNA virus. RdRp is under less immune selection pressure from its host as compared to VP1 because it is not the target of neutralizing antibodies. Thus, this result is not surprising. The relatively high conserved feature of the norovirus GI RdRp region also ensures the replication of norovirus.

The evolutionary rate of the norovirus GI RdRp region was also estimated in our study, and a lower evolutionary rate was observed in the GI RdRp gene when compared with GII RdRp [2]. For each P-genotype of norovirus GI, the evolutionary rate varied. In the recombination strains of norovirus, the acquisition of new polymerase can change the evolutionary rate of VP1 [43]. For example, a new GII.P16 polymerase resulted in a higher evolutionary rate of GII.2 compared with previous GII.P16-GII.2 strains [51]. Then, we compared the evolutionary rate of norovirus GI RdRp with the GI VP1 gene from a previous study, in which the evolutionary rates of GI.2, GI.3, GI.4 and GI.6 were estimated [35]. We found that higher evolutionary rates of GI RdRp indicated higher evolutionary rates of GI VP1, except for GI.P6. This association may be another piece of evidence of the influence of norovirus RdRp on the VP1 gene.

In summary, we found that norovirus GI.P13 was the most dominant P-genotype of norovirus GI in the GenBank database. The norovirus GI RdRp region can be clustered into three groups, and the molecular evolutionary characteristics were various in each P-genotype. Most of the amino acids of the GI RdRp gene were under negative selection, and amino acid substitutions in the GI RdRp gene accumulated slowly over time. Additionally, due to the fact that the human norovirus GI genotype is just less detected and reported, as it tends to cause fewer outbreaks than the GII genotype, there is a limited sample size, which may cause selection bias in this study. To better understand the effect of RdRp on the norovirus activity in the population, a large-scale evolutionary analysis of RdRp and the VP1 gene in the same strain is indispensable, which depends on the surveillance system and availability of sequencing technology.

## Figures and Tables

**Table 1:** Summary and identity of norovirus GI RdRp strains in this study.

Genotype	No. of Strains	Year of Collection	Identity	
			Nucleotide	Amino Acid
GI.P1	33	1968–2018	85.6~100%	96.4~100%
GI.P2	15	1991–2018	88.3~99.9%	98.4~100%
GI.P3	38	1978–2019	87.1~100%	95.6~100%
GI.P4	19	1987–2021	90.5~100%	99.2~100%
GI.P5	2	2008–2012	92.7%	98.2%
GI.P6	10	1997–2013	91.6~100%	98.2~100%
GI.P7	17	1990–2021	86.2~99.9%	97.2~100%
GI.P8	5	2007–2010	97.9~99.9%	99.8~100%
GI.P9	5	2012–2016	98.8~99.8%	99.8~100%
GI.P10	5	1990–2018	88.5~95.2%	98.4~99.4%
GI.P11	30	2006–2021	91.6~100%	98.6~100%
GI.P12	4	2018	99.4~100%	100%
GI.P13	20	1978–2018	87.2~100%	97.4~100%
GI.P14	2	1979–2015	89.9%	98.8%
All	205	1968–2021	70.5~100%	81.2~100%

**Table 2:** Evolutionary parameters of norovirus GI RdRp region.

Genotype	Evolutionary Rate (Substitutions/Site/Year)		The Most Recent Common Ancestor	
	Mean	95% HPDs	Mean	95% HPDs
GI.P1	3.949 × 10^−3^	1.936 × 10^−3^–6.083 × 10^−3^	1955	1934–1968
GI.P2	1.373 × 10^−3^	8.409 × 10^−4^–1.896 × 10^−3^	1947	1921–1969
GI.P3	4.303 × 10^−3^	2.728 × 10^−3^–5.954 × 10^−3^	1963	1941–1979
GI.P4	2.532 × 10^−3^	1.423 × 10^−3^–3.624 × 10^−3^	1977	1959–1987
GI.P6	3.593 × 10^−3^	6.888 × 10^−4^–6.269 × 10^−3^	1987	1967–1997
GI.P7	2.892 × 10^−3^	1.077 × 10^−3^–4.000 × 10^−3^	1966	1920–1990
GI.P11	1.623 × 10^−3^	9.729 × 10^−4^–2.369 × 10^−3^	1981	1958–1999
GI.P13	2.635 × 10^−3^	1.820 × 10^−3^–3.500 × 10^−3^	1950	1966–2009
All	1.821 × 10^−3^	1.294 × 10^−3^–2.344 × 10^−3^	1484	1108–1994

HPDs: highest posterior densities.

## Data Availability

Data sources are available in the GenBank nucleotide database (https://www.ncbi.nlm.nih.gov/genbank/ (accessed on 15 September 2022).

## Supplementary Materials

The following supporting information can be downloaded at: https://www.mdpi.com/article/10.3390/v15010166/s1, Table S1: Strains analyzed in this study; Table S2: Intergenotype mean amino acid distances of norovirus GI RdRp region; Table S3: Mean pairwise phylogenetic distance at intragenotype level; Figures S1–S8: Phylogenetic trees of norovirus GI.P-genotypes with more than 10 strains.
