# Supplementary material for: Molecular Evolution of RNA-Dependent RNA Polymerase Region in Norovirus Genogroup I
Source: Viruses. 2023 Jan 5;15(1):166. doi: 10.3390/v15010166 (PMC9861054; doi:10.3390/v15010166)
Supplement: Supplementary file 1 [file viruses-15-00166-s001.zip › viruses-2072159-supplementary.pdf]

Table S1. Strains analyzed in this study

Accession number	Genotype	Isolation year
M87661	GI.1P[1]	1968
JX023285	GI.1P[1]	1968
KF429765	GI.1P[1]	1968
KF429789	GI.1P[1]	1978
KF429783	GI.1P[1]	1979
KF429774	GI.1P[1]	1973
KF429773	GI.1P[1]	1974
KF429770	GI.1P[1]	1973
KF429761	GI.1P[1]	1972
MZ462933	GI.1P[1]	2016
LC122689	GI.1P[1]	2006
LC122688	GI.1P[1]	2012
MZ220330	GI.1P[1]	2015
MZ220331	GI.1P[1]	2015
MT008453	GI.1P[1]	2018
MT357993	GI.1P[1]	2018
MT357992	GI.1P[1]	2018
MT357991	GI.1P[1]	2018
MH638228	GI.1P[1]	2017
KF039737	GI.1P[1]	2009
KF039736	GI.1P[1]	2010
KF039735	GI.1P[1]	2008
KF039734	GI.1P[1]	2009
KF039733	GI.1P[1]	2011
KF039732	GI.1P[1]	2009
KF039731	GI.1P[1]	2009
KF039730	GI.1P[1]	2011
KF039729	GI.1P[1]	2010
KF039728	GI.1P[1]	2008
KF039727	GI.1P[1]	2009
KF039726	GI.1P[1]	2009
KF039725	GI.1P[1]	2010
MH638229	GI.1P[1]	2017
MW305481	GI.2P[2]	2008
LC122690	GI.2P[2]	2013
MZ223426	GI.2P[2]	2014
MZ223425	GI.2P[2]	2014
MT372465	GI.2P[2]	2017
MT372464	GI.2P[2]	2017
MH218720	GI.2P[2]	2014

MH218656	GI.2P[2]	2015
MH218649	GI.2P[2]	2015
MG599789	GI.2P[2]	2016
KF306212	GI.2P[2]	2013
L07418	GI.2P[2]	1991
FJ515294	GI.2P[2]	2003
MN416764	GI.2P[2]	2018
JN176918	GI.2P[2]	2006
MW305510	GI.3P[3]	1988
MW305509	GI.3P[3]	1988
MW305500	GI.3P[3]	2016
MW305495	GI.3P[3]	2005
MW305494	GI.3P[3]	2004
LC122719	GI.3P[3]	2012
LC122718	GI.3P[3]	2011
LC122717	GI.3P[3]	2014
LC122716	GI.3P[3]	2014
LC122715	GI.3P[3]	2014
MW305490	GI.3P[3]	1988
MW305489	GI.3P[3]	1988
MT089580	GI.3P[3]	2019
MT089579	GI.3P[3]	2019
MT089578	GI.3P[3]	2019
MT372470	GI.3P[3]	2015
MT372468	GI.3P[3]	2015
MT372463	GI.3P[3]	2016
MT031988	GI.3P[3]	2019
MK789654	GI.3P[3]	2014
MN448477	GI.3P[3]	2013
MG557650	GI.3P[3]	2016
MH608286	GI.3P[3]	2014
MH218729	GI.3P[3]	2015
MH218728	GI.3P[3]	2015
MH218727	GI.3P[3]	2015
MH218726	GI.3P[3]	2014
MH218725	GI.3P[3]	2014
MH218724	GI.3P[3]	2014
MH218722	GI.3P[3]	2014
MH218664	GI.3P[3]	2015
MH218659	GI.3P[3]	2015
MH218647	GI.3P[3]	2015
KY934262	GI.3P[3]	2015
NC_039897	GI.3P[3]	2007

KJ196292	GI.3P[3]	2007
KJ194510	GI.3P[3]	1995
JX846929	GI.3P[3]	1978
AB042808	GI.4P[4]	1987
MW305504	GI.4P[4]	2013
MW305499	GI.4P[4]	2015
LC122712	GI.4P[4]	2004
LC122711	GI.4P[4]	2005
LC122710	GI.4P[4]	2010
LC122708	GI.4P[4]	2010
LC122706	GI.4P[4]	2014
LC122705	GI.4P[4]	2013
LC122704	GI.4P[4]	2012
LC122703	GI.4P[4]	2014
OK562729	GI.5P[4]	2021
MZ229462	GI.4P[4]	2012
MZ229330	GI.4P[4]	2012
MN938461	GI.4P[4]	2019
MN938460	GI.4P[4]	2019
LC122709	GI.4P[4]	2010
LC122707	GI.4P[4]	2008
LN854563	GI.4P[4]	2011
MW305487	GI.5P[5]	2008
MW305496	GI.5P[5]	2012
AF093797	GI.6P[6]	1997
MW305503	GI.6P[6]	2013
MW305502	GI.6P[6]	2013
MW305501	GI.6P[6]	2013
LC122702	GI.6P[6]	2013
LC122701	GI.6P[6]	2012
LC122700	GI.6P[6]	2012
LC122699	GI.6P[6]	2012
LC122698	GI.6P[6]	2013
MW305505	GI.6P[6]	2013
MW305508	GI.7P[7]	1990
LC122723	GI.7P[7]	2014
LC122722	GI.7P[7]	2010
LC122721	GI.7P[7]	2009
MT008459	GI.7P[7]	2018
MT372469	GI.7P[7]	2017
MT372462	GI.7P[7]	2014
MT357994	GI.7P[7]	2018
LC646335	GI.7P[7]	2021

LC646334	GI.7P[7]	2020
KX907730	GI.7P[7]	2014
KX907729	GI.7P[7]	2011
KU311161	GI.7P[7]	2012
MW305486	GI.7P[7]	2009
MW305483	GI.7P[7]	2010
MZ215727	GI.7P[7]	2014
LC122720	GI.7P[7]	2007
MW305484	GI.8P[8]	2008
MW305482	GI.8P[8]	2008
MT372476	GI.8P[8]	2010
KJ196298	GI.8P[8]	2007
KP407450	GI.8P[8]	2008
MZ215728	GI.9P[9]	2013
MT372475	GI.9P[9]	2013
MT372474	GI.9P[9]	2013
KF586507	GI.9P[9]	2012
KX907731	GI.9P[9]	2016
MW305507	GI.3P[10]	1990
MW305506	GI.3P[10]	1990
ON033826	GI.3P[10]	2018
LC122713	GI.3P[10]	2012
MT008457	GI.3P[10]	2018
MN226991	GI.6P[11]	2018
MW243609	GI.6P[11]	2019
MW305498	GI.6P[11]	2013
MW305497	GI.6P[11]	2013
LC122697	GI.6P[11]	2013
LC122696	GI.6P[11]	2010
LC122695	GI.6P[11]	2009
LC122694	GI.6P[11]	2006
LC122693	GI.6P[11]	2010
LC122692	GI.6P[11]	2008
LC122691	GI.6P[11]	2013
MZ227265	GI.6P[11]	2016
MZ227264	GI.6P[11]	2016
MT372477	GI.6P[11]	2018
MT357996	GI.6P[11]	2018
MT357995	GI.6P[11]	2018
MK789656	GI.6P[11]	2014
MK789655	GI.6P[11]	2014
MK236611	GI.6P[11]	2016
MK236610	GI.6P[11]	2016

MK236609	GI.6P[11]	2016
MK236608	GI.6P[11]	2016
KP407451	GI.6P[11]	2008
LC726062	GI.6P[11]	2021
LC726061	GI.6P[11]	2021
LC726060	GI.6P[11]	2021
LC646339	GI.6P[11]	2021
LN854564	GI.6P[11]	2012
JQ388274	GI.6P[11]	2010
JN176919	GI.6P[11]	2007
MT008460	GI.5P[12]	2018
MT008458	GI.5P[12]	2018
MT008454	GI.5P[12]	2018
MH443711	GI.5P[12]	2018
MW305493	GI.3P[13]	1978
MW305492	GI.3P[13]	1978
MZ462932	GI.3P[13]	2017
MZ462930	GI.3P[13]	2016
MZ462929	GI.3P[13]	2016
LC122714	GI.3P[13]	2012
MZ203488	GI.3P[13]	2012
MW445537	GI.3P[13]	2018
MT008456	GI.3P[13]	2018
MT008455	GI.3P[13]	2018
MT526277	GI.3P[13]	2014
MT526276	GI.3P[13]	2014
MT372467	GI.3P[13]	2015
MT372466	GI.3P[13]	2015
MH218723	GI.3P[13]	2014
MH218721	GI.3P[13]	2014
MK073893	GI.3P[13]	2016
MK073892	GI.3P[13]	2016
MK073887	GI.3P[13]	2016
JQ911594	GI.3P[13]	2010
AB187514	GI.3P[14]	1979
MH218730	GI.3P[14]	2015

Table S2. Intergenotype mean amino acid distances of norovirus GI RdRp region

	GI.P1	GI.P2	GI.P3	GI.P4	GI.P5	GI.P6	GI.P7	GI.P8	GI.P9	GI.P10	GI.P11	GI.P12	GI.P13
GI.P1													
GI.P2	0.092383233												
GI.P3	0.191530727	0.1815406											
GI.P4	0.111150298	0.09680699	0.181585522										
GI.P5	0.126142456	0.10581379	0.193193802	0.0444151									
GI.P6	0.116252778	0.11426593	0.181527819	0.0688966	0.07347037								
GI.P7	0.192033412	0.16729053	0.08622667	0.1700853	0.17604619	0.17142439							
GI.P8	0.184398246	0.16970644	0.034081092	0.174435	0.19357631	0.18351606	0.085287142						
GI.P9	0.18153877	0.16236691	0.086117038	0.1766757	0.18757388	0.1779519	0.045783114	0.0870121					
GI.P10	0.19501469	0.1795398	0.048884551	0.1778744	0.18853426	0.18189678	0.071624918	0.0429784	0.08140205				
GI.P11	0.090008051	0.01970087	0.168790057	0.0953761	0.1020701	0.11933444	0.15935787	0.1627544	0.1503788	0.174041197			
GI.P12	0.126378406	0.10184267	0.191577521	0.0409281	0.01905417	0.07048619	0.176043386	0.1923724	0.18518325	0.186618083	0.10206044		
GI.P13	0.188105642	0.17711825	0.038558787	0.1819061	0.19502427	0.17718289	0.084026729	0.0346242	0.08632318	0.047151278	0.170112538	0.19141255	
GI.P14	0.198479986	0.18328871	0.038608293	0.1810736	0.19550693	0.18709684	0.075499461	0.0367987	0.07667803	0.033513102	0.170101212	0.191893897	0.04194652

Table S3. Mean pairwise phylogenetic distance at intragenotype level

Genotype	Mean	Standard deviation
P1	0.1690828	0.1662596
P2	0.07239842	0.06385872
P3	0.1182628	0.06035648
P4	0.06680355	0.04665033
P6	0.03132472	0.03601332
P7	0.1187966	0.06063878
P11	0.05030093	0.03110564
P13	0.092069	0.08904469

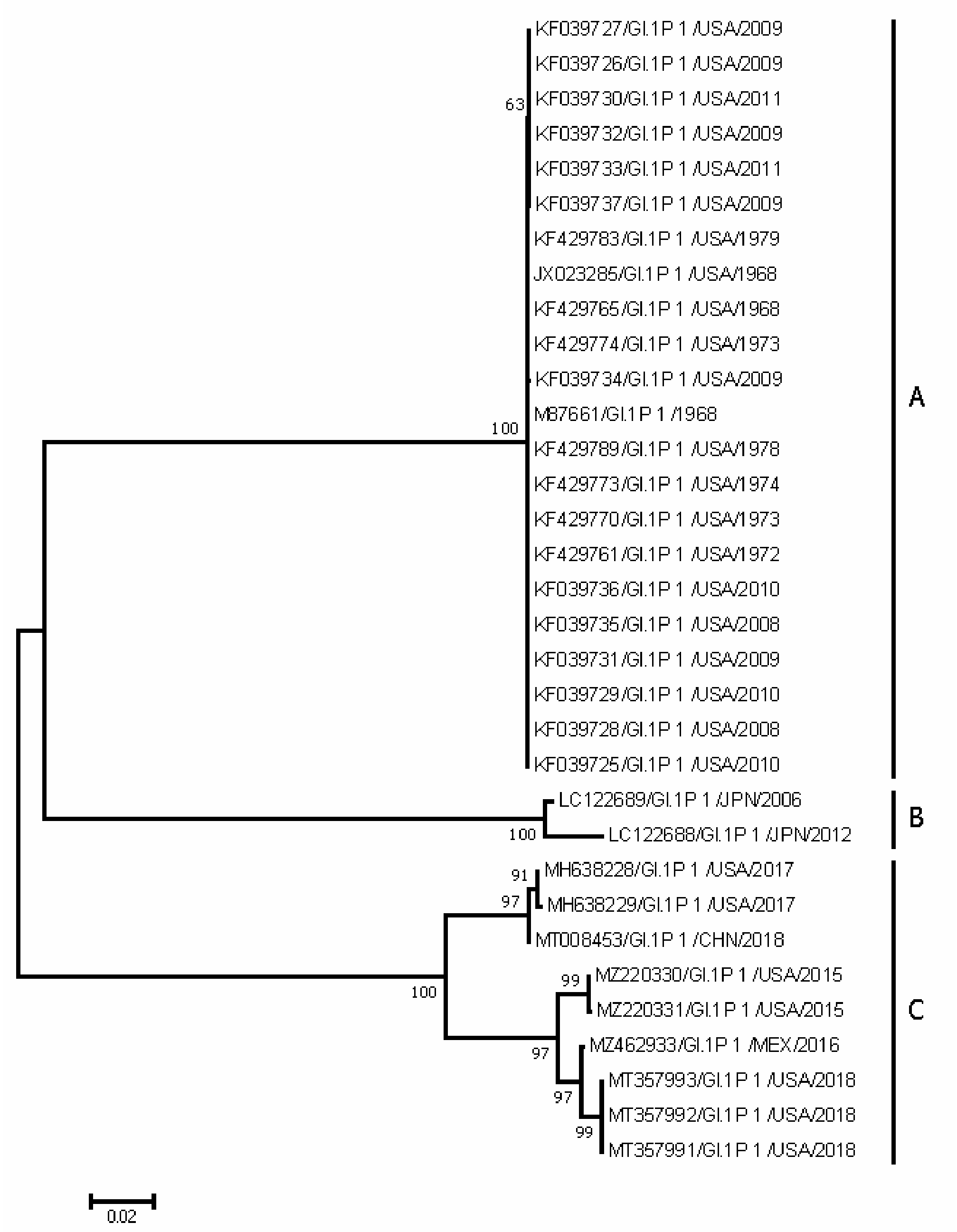

Figure S1. Phylogenetic tree of GI.P1

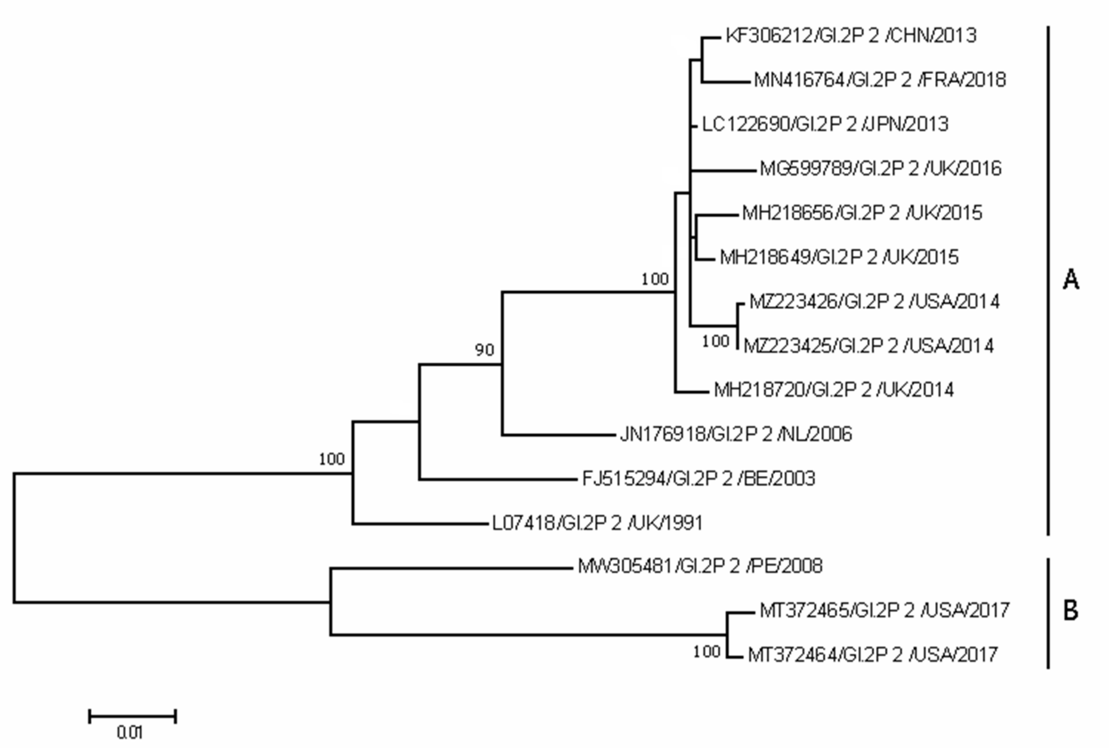

Figure S2. Phylogenetic tree of GI.P2

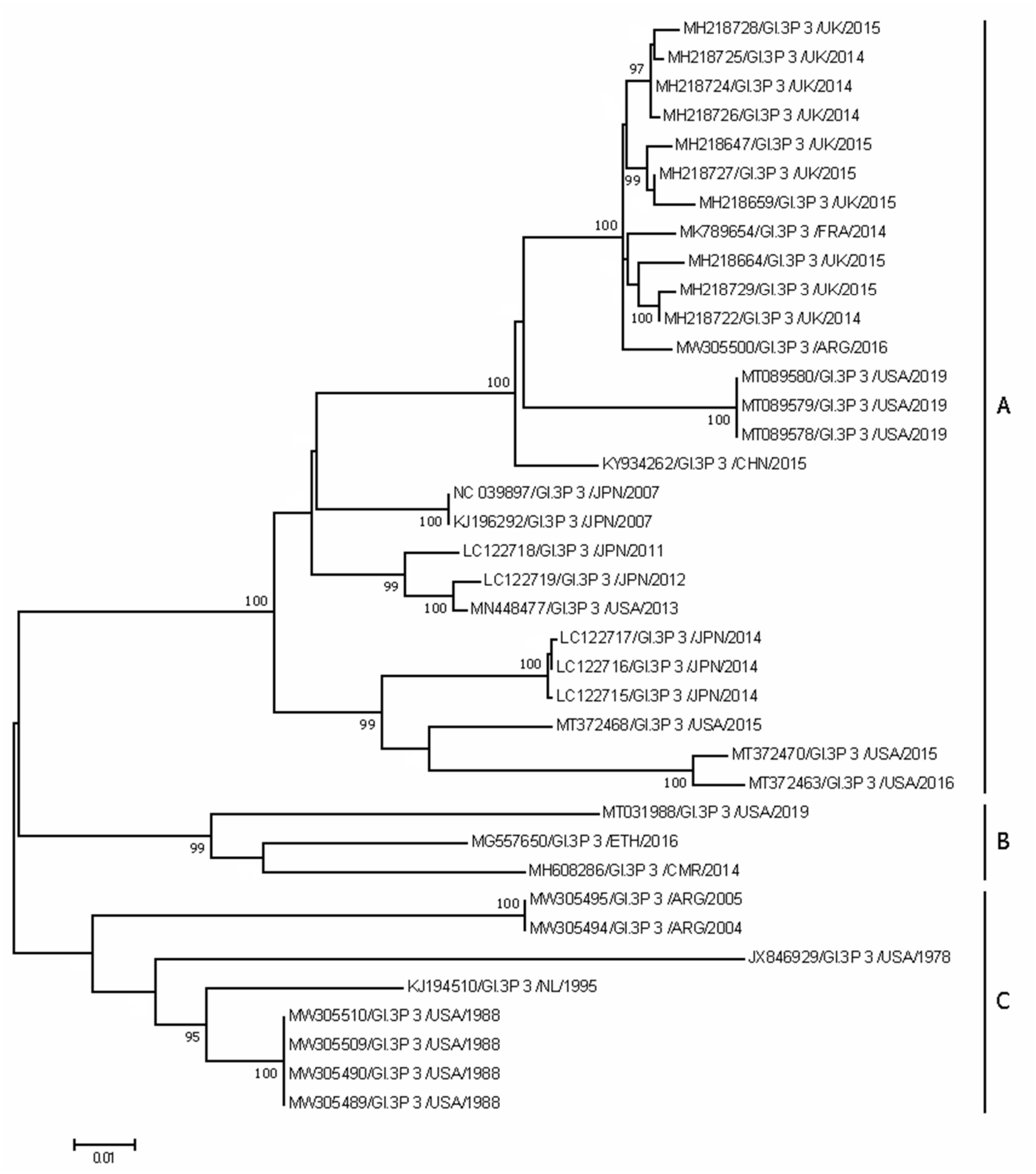

Figure S3. Phylogenetic tree of GI.P3

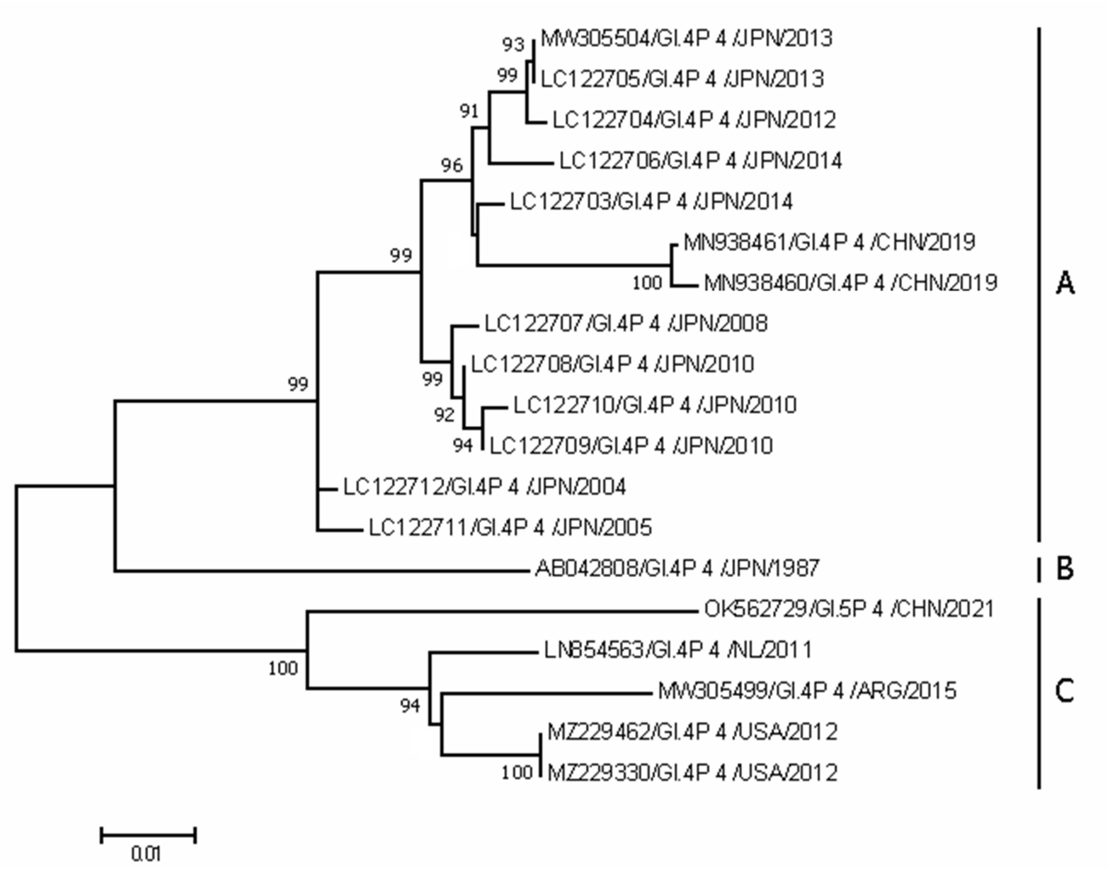

Figure S4. Phylogenetic tree of GI.P4

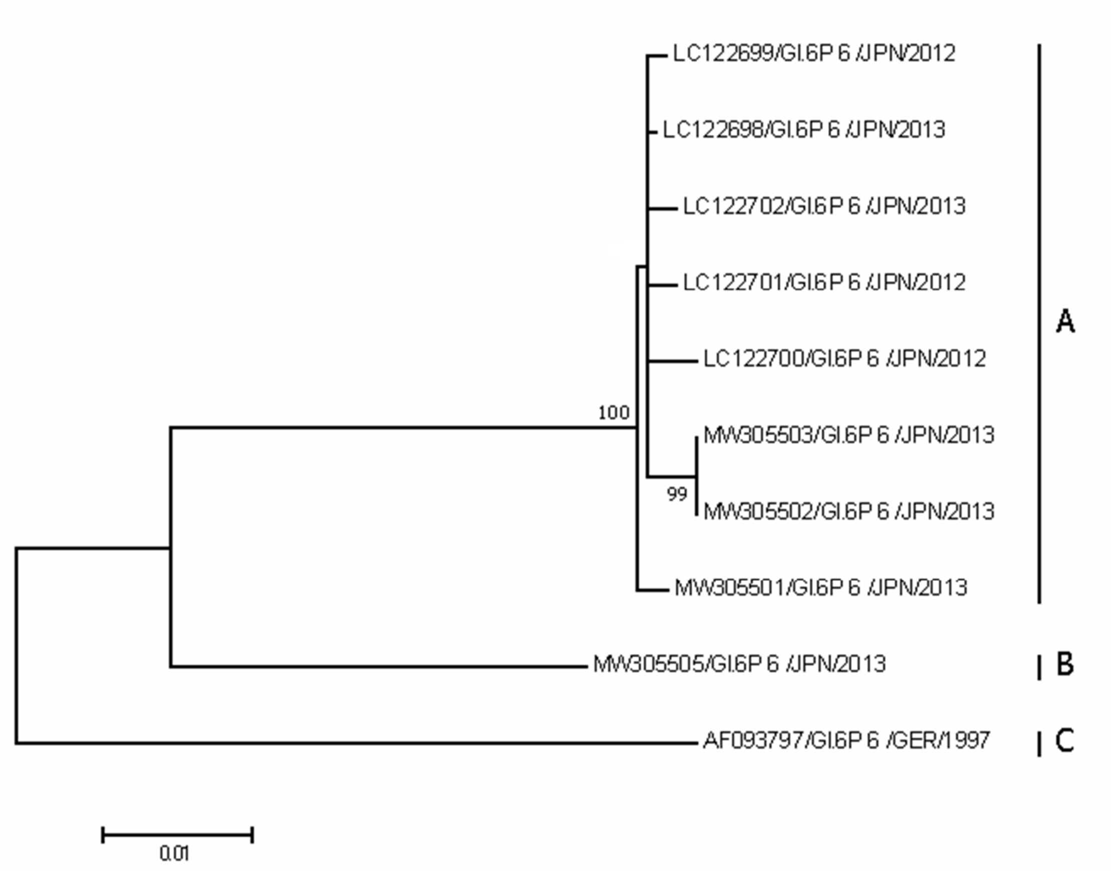

Figure S5. Phylogenetic tree of GI.P6

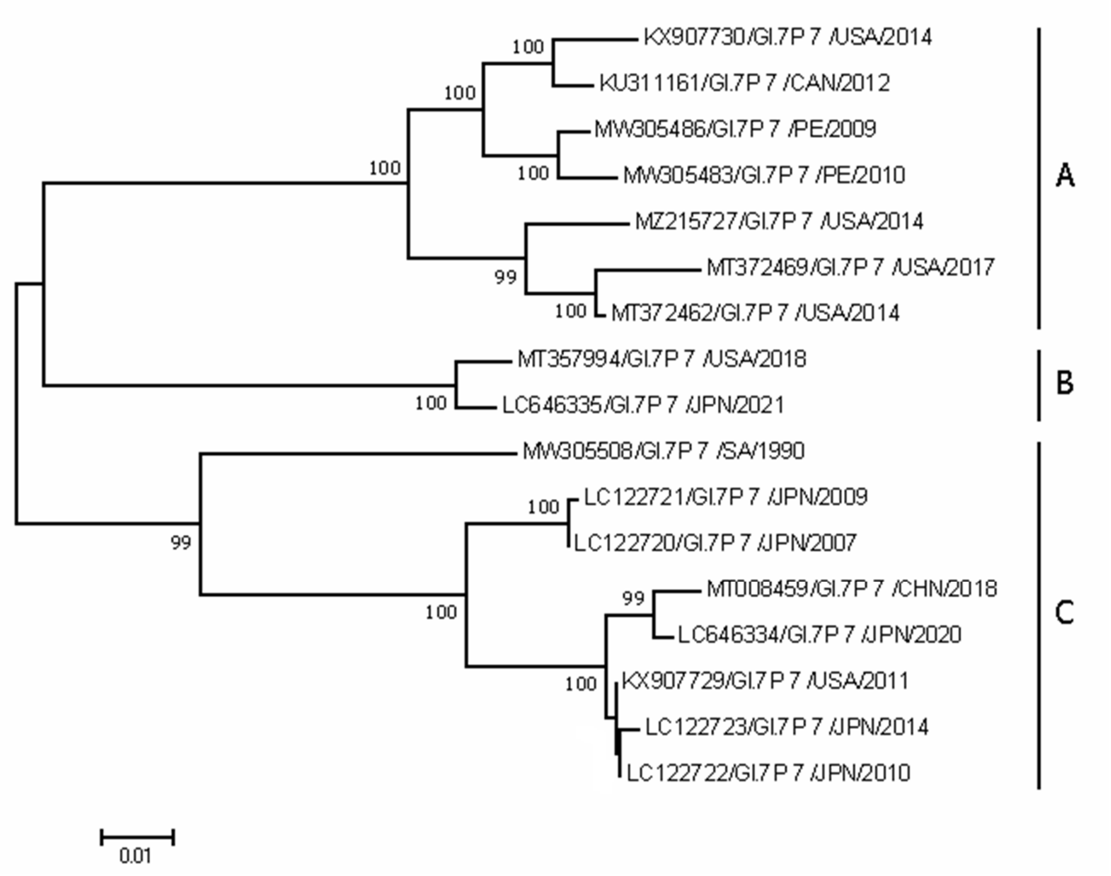

Figure S6. Phylogenetic tree of GI.P7

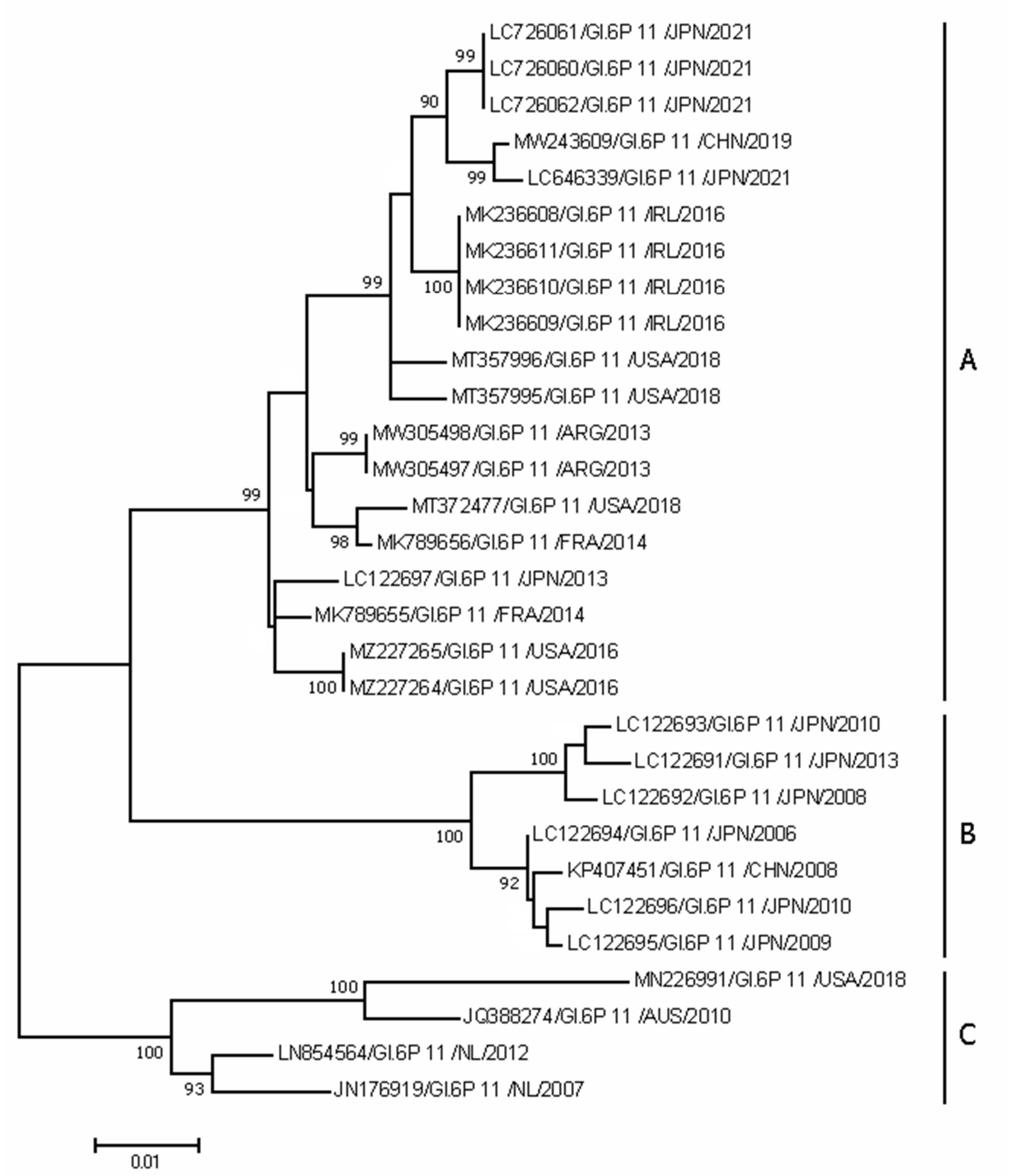

Figure S7. Phylogenetic tree of GI.P11

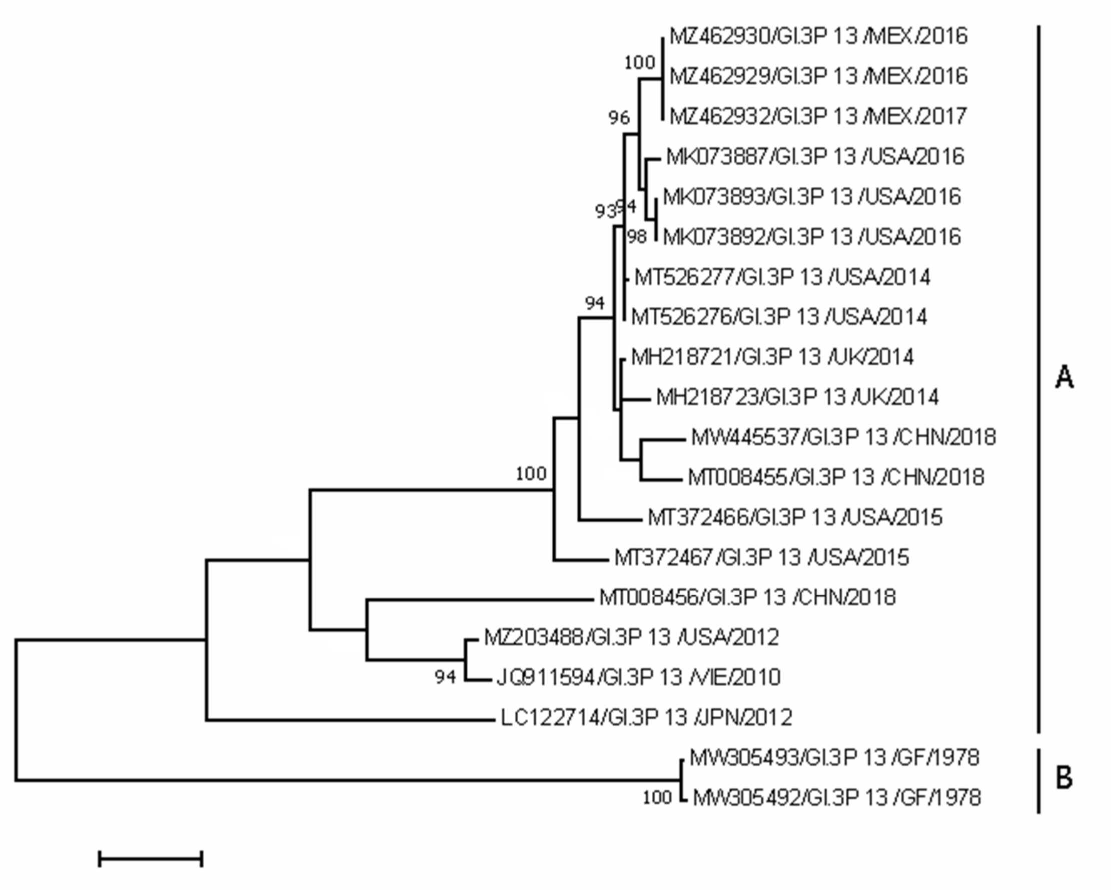

Figure S8. Phylogenetic tree of GI.P13
